# Cell signaling pathways in autosomal-dominant leukodystrophy (ADLD): the intriguing role of the astrocytes

**DOI:** 10.1007/s00018-020-03661-1

**Published:** 2020-10-09

**Authors:** Stefano Ratti, Isabella Rusciano, Sara Mongiorgi, Eric Owusu Obeng, Alessandra Cappellini, Gabriella Teti, Mirella Falconi, Lia Talozzi, Sabina Capellari, Anna Bartoletti-Stella, Pietro Guaraldi, Pietro Cortelli, Pann-Ghill Suh, Lucio Cocco, Lucia Manzoli, Giulia Ramazzotti

**Affiliations:** 1 Cellular Signalling Laboratory, Department of Biomedical and NeuroMotor Sciences (DIBINEM), University of Bologna, Bologna, Italy; 2 Department of Biomedical and NeuroMotor Sciences (DIBINEM), University of Bologna, Via Irnerio 48, Bologna, Italy; 3 Functional MR Unit, Department of Biomedical and NeuroMotor Sciences (DIBINEM), University of Bologna, Bologna, Italy; 4 Department of Biomedical and NeuroMotor Sciences (DIBINEM), University of Bologna, Bologna, Italy; 5 IRCCS Istituto delle Scienze Neurologiche di Bologna, UOC NeuroMet, Bologna, Italy; 6 Korea Brain Research Institute, Daegu, Republic of Korea; 7 School of Life Sciences, UNIST, Ulsan, Republic of Korea

**Keywords:** Lamin B1, Cellular signaling, ADLD, LIF, Astrocyte

## Abstract

Autosomal-dominant leukodystrophy (ADLD) is a rare fatal neurodegenerative disorder with overexpression of the nuclear lamina component, Lamin B1 due to LMNB1 gene duplication or deletions upstream of the gene. The molecular mechanisms responsible for driving the onset and development of this pathology are not clear yet. Vacuolar demyelination seems to be one of the most significant histopathological observations of ADLD. Considering the role of oligodendrocytes, astrocytes, and leukemia inhibitory factor (LIF)-activated signaling pathways in the myelination processes, this work aims to analyze the specific alterations in different cell populations from patients with LMNB1 duplications and engineered cellular models overexpressing Lamin B1 protein. Our results point out, for the first time, that astrocytes may be pivotal in the evolution of the disease. Indeed, cells from ADLD patients and astrocytes overexpressing LMNB1 show severe ultrastructural nuclear alterations, not present in oligodendrocytes overexpressing LMNB1. Moreover, the accumulation of Lamin B1 in astrocytes induces a reduction in LIF and in LIF-Receptor (LIF-R) levels with a consequential decrease in LIF secretion. Therefore, in both our cellular models, Jak/Stat3 and PI3K/Akt axes, downstream of LIF/LIF-R, are downregulated. Significantly, the administration of exogenous LIF can partially reverse the toxic effects induced by Lamin B1 accumulation with differences between astrocytes and oligodendrocytes, highlighting that LMNB1 overexpression drastically affects astrocytic function reducing their fundamental support to oligodendrocytes in the myelination process. In addition, inflammation has also been investigated, showing an increased activation in ADLD patients’ cells.

## Introduction

Autosomal-dominant leukodystrophy (ADLD) is an extremely rare, fatal, and late onset progressive neurological disorder which affects the white matter of the central nervous system (CNS) usually, in the IV or V decade [1–3]. The real prevalence of this ultra-rare pathology remains still uncertain [4] with sporadic new clinical case reports from different geographical areas, suggesting a possible heterogeneity in the first clinical manifestations and signs [5–10]. In majority of ADLD cases, the first clinical manifestations are related to autonomic dysfunction from bladder or bowel dysfunction to orthostatic hypotension, temperature dysregulation, and anhidrosis [11–15]. Usually, pyramidal and cerebellar dysfunction, muscle weakness, and spasticity appear after the autonomic dysfunction and before the cognitive impairment [1, 2, 16, 17]. ADLD is fatal and up to now, the treatment is only symptomatic: patients survive 1 or 2 decades after the disease onset [12]. The diagnosis of ADLD is supported by the combination of patient’s clinical history, physical examinations, magnetic resonance imaging (MRI) showing symmetrical cerebral white matter hyperintensities from the motor cortex to the medulla oblongata and involvement of the upper and middle cerebellar peduncles and confirmed by Lamin B1 (LMNB1) gene analysis [1, 13, 16, 18]. Histopathological analysis of ADLD brains underline the vacuolar demyelination processes [18–20]. Genetically, ADLD is characterized by alterations of the LMNB1 gene (chr5q23.2), resulting in the overexpression of Lamin B1, a component of the nuclear lamina [1, 9, 21]. At least two genetic alterations have been identified: LMNB1 gene tandem duplication and LMNB1 gene upstream deletions involving its regulatory sequences [8] [13]. Indeed, the molecular aspects detailing how the genetic alterations affect the cellular mechanisms to drive the onset and development of this pathology are not clear yet. Demyelination seems to be one of the most significant aspects of ADLD. Alterations in the myelin or in the myelination process, or both, due to inflammatory, toxic, or genetic etiology can trigger severe kind of neurological disorders. The understanding of the primary cause is essential to find and target the correct altered mechanism [22]. Oligodendrocytes in the CNS produce myelin by wrapping their plasma membranes spirally around axons or nerve fibers and are crucial for providing metabolic support to neurons [23]. On the other hand, astrocytes provide for homeostasis and defense of the CNS and are essential for adaptive plasticity that defines the functional maintenance of CNS in the development and aging [24]. Among the signaling molecules studied in the CNS, the leukemia inhibitory factor (LIF), a member of the interleukin-6-type cytokine family, has been classified as a pro-myelinating factor since it has been shown that LIF signaling between astrocytes and oligodendrocytes regulates myelination in response to electrical activity in axons [25]. LIF signaling is triggered by LIF binding to its receptor (LIF-R) that is associated to the transmembrane protein gp130, determining the activation of the Janus kinase–signal transducer and activator of transcription protein 3 (Jak/Stat3), phosphatidylinositol-3 phosphate kinase (PI3K)/Akt- and mitogen-activated protein kinase kinase (MEK)/ERK signaling pathways [26]. The correlation between the mammalian target of rapamycin complex 1 (mTORC1), a mediator belonging to these signaling pathways, and myelination has been demonstrated in relation to the metabolic function required in myelin formation both in CNS and in peripheral nervous system [27–31].

Indeed, inflammation has been shown to be involved in several neurodegenerative processes [32] and Lamin B1 accumulation has been linked to both nuclear structural changes [33] and senescence [34] in relation to several stress conditions, even if its role is still controversial [35–38]. Moreover, considering the pivotal role of Lamin B1 in nuclear structure maintenance [39], in myelination in the CNS [40] and dendrite development [41], the understanding of the molecular mechanisms affected by Lamin B1 alteration in specific tissues and pathologies is requested. The present work suggests novel signaling pathways and morphological changes related to Lamin B1 accumulation and to ADLD disease development.

## Materials and methods

### Cell culture and lentiviral transduction

Human glioblastoma astrocytic cell line U87-MG (HTB-14 ATCC, Old Town Manassas, Virginia, US) was cultured in Eagle's Minimum Essential Medium (EMEM) (Corning, New York, US) with 10% FBS and 1% Penicillin/Streptomycin (Sigma-Aldrich, St. Louis, MO, US). Human oligodendrocytic cell line MO3.13 (Cedarlane Laboratories, Burlington, Canada) was cultured in Dulbecco’s modified Eagle medium (DMEM) without sodium pyruvate (Corning) supplemented with 10% FBS and 1% Penicillin/Streptomycin.

Human embryonic kidney HEK 293 T cells (Genecopoeia Inc, US) were cultured in DMEM (Corning) with 10% FBS and 1% Penicillin/Streptomycin; however, 5% FBS was used during transfection.

Primary human fibroblasts isolated from skin biopsies of six ADLD patients carrying LMNB1 gene duplication and six non-diseased donors were obtained from the IRCCS Istituto delle Scienze Neurologiche di Bologna, UOC NeuroMet, Italy. This study was approved by the AUSL Bologna Ethical Committee and informed consents were obtained from all participants. Human primary fibroblasts were grown in DMEM supplemented with 10% FBS and 1% Penicillin/Streptomycin.

All cells were maintained in a humidified incubator at 37 °C with 5% CO_2_.

EX-I3724-Lv201 coding for Homo Sapiens LMNB1 and EX-NEG-Lv201 empty control vectors (Genecopoeia) were used to construct lentiviruses used to overexpress both LMNB1 and green fluorescent protein (GFP) for our disease model as well as lentiviruses coding only for GFP, as control. The Lenti-Pac HIV expression packaging kit (Genecopoeia) was used as the viral packaging system to transfect HEK293T cells according to manufacturer’s protocol. The supernatants containing the viruses were harvested 24–48 h after transfection and filtered through a 0.45 mm cellulose acetate filter. To perform viral transduction, U87-MG and MO3.13 cells were plated the day before infection, respectively, at 5 × 10^5^ cells/well and 8 × 10^5^ cells/well of a 6-well plate. The next day virus supernatants were added with polybrene 8 µg/ml for U87-MG and 5 µg/ml for MO3.13 cells and applied to cultures of target cells. The supernatants were replaced with fresh media the next day and 48 h after transduction, cells were selected for 48 h in growth media supplemented with 2 µg/ml of puromycin (Sigma-Aldrich) for U87-MG cells and 1 µg/ml of puromycin for MO3.13 cells.

To test leukemia inhibitory factor (LIF) ability to induce a normal phenotype rescue, U87-MG and MO3.13 cells were treated with 80 ng/ml of Recombinant Human LIF (Thermo Fisher Scientific, Waltham, MA, US) 48 h post-transduction for 48 h (96 h in total) [42].

### RNA extraction, reverse transcription, and real-time PCR

RNeasy Mini Kit (Qiagen, Hilden, Germany) was used to extract total RNA from samples. The Nanodrop spectrophotometer was used to quantify extracted RNA. Using the iScript gDNA Clear cDNA Synthesis Kit (Bio-Rad, Hercules, CA, US), 1 µg of total RNA was reverse transcribed into cDNA following the manufacturer’s protocol. mRNA expression levels were detected using a TaqMan probe-based real-time PCR system (Thermo Fisher Scientific). Real-time PCR was performed on 100 ng of cDNA per well, with the ABI PRISM 7300 real-time PCR machine (Applied Biosystems, Life Technologies, Carlsbad, CA, US). *GAPDH* was used as the housekeeping gene and data are presented as fold changes relative to expression levels of control samples in accordance with the 2^−ΔΔCT^ formula. Validated gene probes used are as follows: LMNB1 Hs.PT.58.40133522, *LIF* Hs.PT.58.27705899, *LIF-R* Hs.PT.58.2980475, *GAPDH* Hs.PT.39a.22214836 (IDT, Coralville, IA, US).

### Protein extraction and western blot

Cells were lysed with T-PER lysis buffer supplemented with Halt protease and phosphatase inhibitor cocktails (all from Thermo Fisher Scientific). Lysed cells were sonicated in just 1 cycle of 15 s duration and at a power of 40–50%. Whole cell lysates were quantified with the Bradford Protein Assay (Bio-Rad). 40 µg of total proteins were separated on bolt 4–12% polyacrylamide-0.1% commercial SDS gels (Thermo Fisher Scientific) and transferred onto nitrocellulose membrane. Membranes were washed with PBS-0.1% Tween-20 (PBST) and non-specific binding sites were blocked with blocking buffer (5% w/v non-fat dry milk in PBST) for 1 h at room temperature. Lastly, membranes were incubated with primary antibodies overnight at 4 °C. The antibodies used were diluted 1:1000 in either bovine serum albumin (BSA) (Sigma-Aldrich) or milk following manufacturer’s protocols. Membranes were washed again with PBST, then incubated with peroxidase conjugated secondary antibodies (Thermo Fisher Scientific) diluted in PBST for 1 h at room temperature. ECL enhanced chemiluminescence reagents (Thermo Fisher Scientific) were used to detect immunoreactive bands and images captured with the ChemiDoc-It^®^ 2Imager digital system (UVP, Upland, CA US).

### Immunocytochemistry

Cells were fixed in ice-cold 100% methanol (Sigma-Aldrich) for 15 min at -20 °C. After blocking in 1% BSA for 1 h at room temperature, cells were incubated with primary antibody overnight at 4 °C. Dilutions of primary antibodies were in accordance with the manufacturer’s instructions. Cells were then incubated in the dark at room temperature for 1 h with corresponding secondary antibodies, Anti-Mouse IgG F(ab')_2_ Fragment antibody conjugated to Alexa Fluor 488 (Cell Signaling Technology, Danvers, MA, US) or Anti-Rabbit IgG F(ab′)_2_ fragment-Cy3 antibody (Sigma-Aldrich). Lastly, nuclei were stained with ProLong Gold Antifade reagent with DAPI (Invitrogen, Thermo Fisher Scientific). Slides were then examined under a Zeiss Axio-Imager Z1 fluorescent microscope (Carl Zeiss International, Germany). At least five different fields were analyzed at 20 × magnification.

### Antibodies

The following antibodies were used in western blotting and immunofluorescence**:** Raptor (CST 2280), PI3K p110α (CST 4249), PI3K p110γ (CST 5405), phospho-Stat3 (CST 9134), phospho-NFkB (CST 3033), phospho-Akt (CST 9271), phospho-p44/42 MAPK (Erk1/2) (CST 4376), phospho-GSK3α/β (CST 8566) from Cell Signaling Technology (Danvers, MA, US). PKCα (PA517551), Lamin B1 (10H34L18), LIF (PA5-21,122), and phospho-Stat4 (71–7900) from Invitrogen, Thermo Fisher Scientific. β-Tubulin (T7816) from Sigma-Aldrich.

### Enzyme‐linked solid phase immunosorbent (ELISA) assay

The concentration of human LIF in growth medium of cultured cells was detected using the Human LIF ELISA Kit assay (Invitrogen, Thermo Fisher Scientific). Briefly, 48 h after transduction cells were puromycin selected for 48 h and 2.5 × 10^5^ cells were seeded per well in a 12-well plate. The next day, supernatants were collected and subsequently centrifuged to eliminate debris (10 min at 6000 g). A 1:2 dilution was created for each supernatant before assaying in triplicate wells following the manufacturer’s instructions. For each sample (wild type, empty vector and LMNB1 overexpressing cells), three supernatants obtained from three separate experiments were assayed. Glomax Discover (Promega, Madison, Wisconsin, US) was used to read absorbance at 450 nm (primary wavelength) and at 620 nm (reference wavelength). Analyzed data are presented as mean ± standard deviation.

### Transmission electron microscopy (TEM) analysis

U87-MG, MO3.13 cells, and primary fibroblasts cultured from skin biopsies of healthy subjects and ADLD patients were fixed with 2.5% glutaraldehyde in 0.1 M cacodylate buffer, for 2 h at 4 °C and then, post fixed in 1% OsO_4_ in 0.1 M cacodylate buffer, for 30 min at room temperature. After few washes in 0.15 M cacodylate buffer, samples were dehydrated through graded acetone solutions and embedded in Epoxy resin (Sigma-Aldrich). Sections of 100 nm each were collected in nickel grids, counterstained with 3% uranyl acetate and 1% lead citrate and observed by Philips CM10 TEM (FEI Company, Eindhoven, The Netherlands), at an accelerating voltage of 80 kV. Images were recorded by Megaview III digital camera (FEI Company).

### Quantitative analysis of misshaped nuclei

Quantitative analysis of misshaped nuclei compared to regular nuclei was carried out on primary fibroblasts isolated from skin biopsies of healthy donors and ADLD patients, grown on cover glasses and processed for immunofluorescence labelling, as described above. Misshaped nuclei were defined by the presence of irregular lining of the nuclear membrane forming nuclear blebs, extensive lobulations, C like and creasing invaginations and stacked nuclear membrane. Nuclei were considered to be normal when ovoid or round shaped with a regular lining nuclear membrane. Quantitative analysis was performed on 100 nuclei of cultured fibroblasts from each patient, at 60X magnification. The number of misshaped nuclei was compared to regular nuclei. Results were expressed as average value (± SD), in percentage, and they were graphically represented for healthy donors and ADLD patients.

### Reactive oxygen species (ROS) production assessment

Intracellular reactive oxygen species (ROS) production was monitored using the cell-permeant probe 2′-7′-dichlorofluorescein (H2DCFDA) in primary dermal fibroblasts. H2DCFDA is a nonfluorescent reduced form of fluorescein, which becomes fluorescent upon cleavage by intracellular esterases and oxidation by ROS. The fluorescence was detected using GloMax® Discover Microplate Reader set on 475 nm (blue) as excitation wavelength and 500–550 nm as emission wavelength.

Briefly, the probe was dissolved in DMSO to obtain a 5 mM solution, kept at − 20 °C and aliquoted in amber micro-tubes to protect it from light-induced oxidation, until the preparation of the fresh 5 µM ready-to-use solution. 4.5 × 10^5^ fibroblasts/sample were washed twice and resuspended in 450 µl of PBS. 150 µl of the sample was used to assess its autofluorescence, the remaining cells were incubated for 10 min at 37 °C with 5 µM H2DCFDA, to load the fluorescent probe. After incubation, 150 µl of fibroblast suspension was left untreated to measure basal ROS production, while 150 µl was treated with 300 µM H_2_O_2_ to evaluate the oxidative activity in response to a stimulus. 50 µl of fibroblasts suspension was seeded per well in a black 96-well plate. Therefore, for each sample, autofluorescence, basal ROS production, and ROS production following H_2_O_2_ treatment were measured in triplicate. For the time course, florescence was read every 15 min for 7 h, after brief shaking.

### Statistical analysis

Statistical analysis was carried out using Graph Pad Prism 5.0 software (San Diego, CA, US) by applying the two-way ANOVA and Sidak post-test. The differences were considered significant with *p* < 0.05* and *p* < 0.01 **.

## Results

### In vitro models: astrocytic and oligodendrocytic cell lines

#### Lamin B1 overexpression affects nuclear morphology in U87-MG cells

**Fig. 1:**
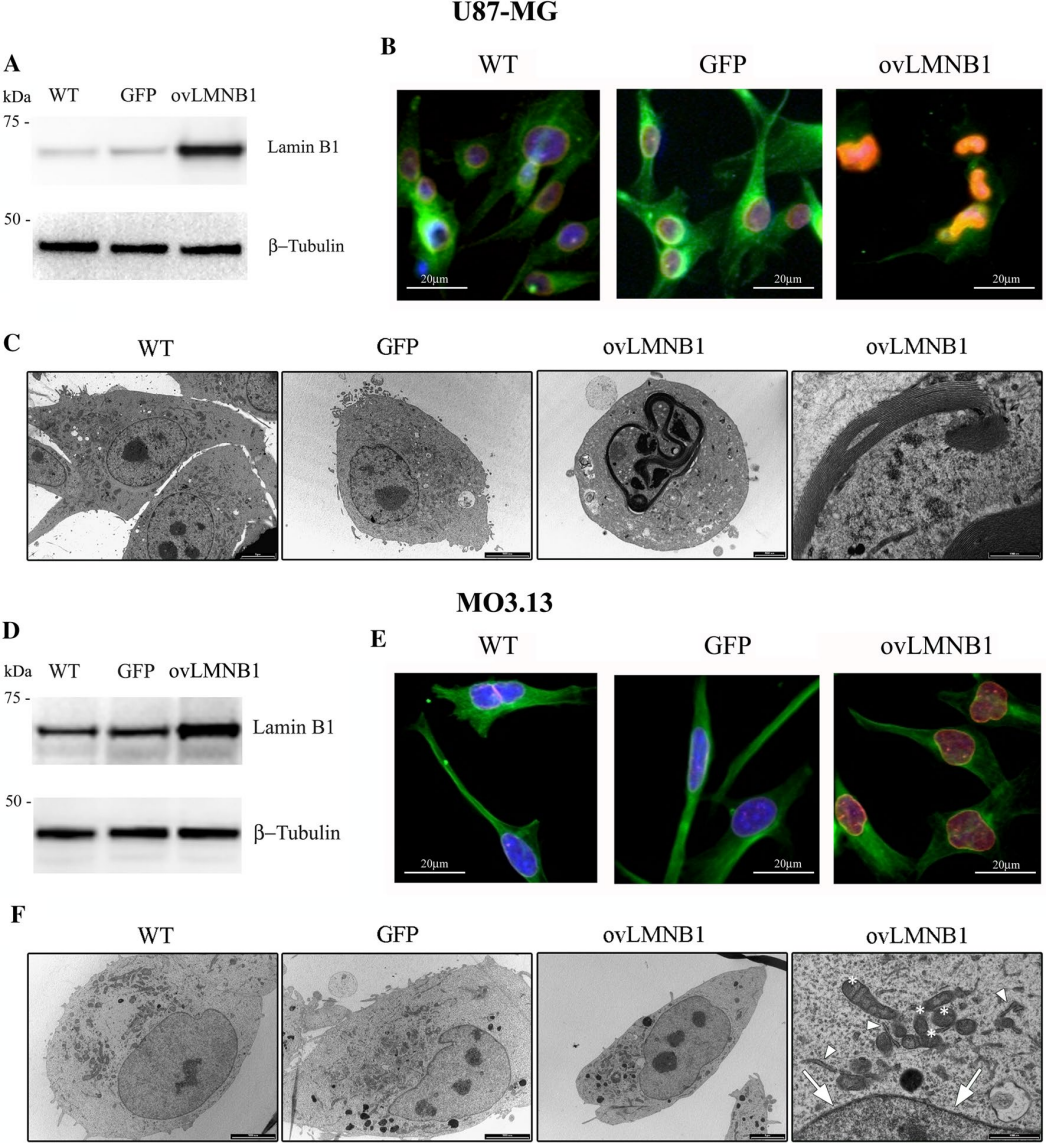
Lamin B1 overexpression affects nuclear morphology in U87-MG cells. Following transduction, Lamin B1 protein levels and localization, and cell morphology were evaluated. Cells transduced with Lamin B1-coding vector (**ovLMNB1**) were compared to wild type (**WT**) and mock-transduced cells (**GFP**). Panels **a** and **d** show western blot analysis of Lamin B1 expression in U87-MG and MO3.13 cells, respectively, using β-tubulin as loading control. Western blot results are representative of three independent experiments. Panels **b** and **e** display immunofluorescence staining of Lamin B1 (red) and β-tubulin (green), in U87-MG and MO3.13 cells, respectively (bar: 20 µM). Nuclei were stained using DAPI (blue). Results are representative of at least five different fields. Panel **c** shows TEM analysis of U87-MG cells. Wild-type U87-MG cells (**WT**) show polygonal cell shape and a round-shaped nucleus (bar: 5 μm). Mock-transduced U87-MG cells (**GFP**) show regular cellular and nuclear shape (bar: 5000 nm). In U87-MG cells overexpressing Lamin B1 (**ovLMNB1**) a misshaped nucleus is observed (bar: 2000 nm) and the nuclear envelope shows several stacked membranes (bar: 1000 nm). Panel **f** displays TEM analysis of MO3.13 cells. Wild-type (**WT**) and mock- transduced (**GFP**) MO3.13 cells show regular cell morphology and nuclear shape (bar: 5000 nm). MO3.13 cells overexpressing Lamin B1 (**ovLMNB1**) show regular nuclear morphology (bar: 5 μm) and no lamellar stacked membranes (arrow), as displayed in the detail of the nuclear envelope. Several mitochondria (*) and regular endoplasmic reticulum (arrowhead) are visible (bar: 1000 nm). The results are representative of three independent experiments

To study the effect of Lamin B1 overexpression on astrocytes and oligodendrocytes, we transiently transduced two cell lines U87-MG and MO3.13, the first being the astrocytic and the second the oligodendrocytic cell line. After puromycin selection, cells were tested for Lamin B1 protein expression and compared to wild type and to cells transduced with an empty vector coding only for GFP (Fig. 1a, d). In addition, Lamin B1 nuclear localization was evaluated by immunofluorescence (IF) (Fig. 1b, e) and nuclear shape was examined by transmission electron microscopy (TEM) (Fig. 1c, f).

Following transduction, western blot analysis revealed that both transduced cell lines show high levels of Lamin B1 and that the increase in Lamin B1 protein level was greater in U87-MG than in MO3.13 cells (Fig. 1a, d). Next, we evaluated if the subcellular localization of Lamin B1 changed in transduced cells compared to controls. We determined that Lamin B1 was localized in the nucleus, particularly in the nuclear lamina and nucleoplasm, both in transduced U87-MG and in MO3.13 cells (Fig. 1b, e). Moreover, IF revealed an increased immunoreactivity in transduced cells overexpressing Lamin B1 compared to controls (WT and GFP) and this confirmed the different levels of overexpression reached in the two cell line models, the astrocytic cell line (U87-MG) being the one showing higher levels of Lamin B1 overexpression. Furthermore, IF also showed alterations in the nuclear shape of U87-MG cells following Lamin B1 accumulation. Therefore, we further investigated nuclear morphology by transmission electron microscopy (TEM) and evaluated the variation in nuclear shape due to Lamin B1 accumulation both in U87-MG (Fig. 1c) and in MO3.13 cell lines (Fig. 1f).

TEM analysis of U87-MG cells overexpressing Lamin B1 showed a round-shaped morphology with misshaped and folded nuclei. At higher magnification, the nuclear envelope appeared to be composed of several stacked membrane layers surrounding the chromatin, which is highly condensed in few masses. Wild type and GFP- expressing cells showed a polygonal shape, with round nuclei and well-preserved chromatin and nucleoli. No lamellar stacked membrane was observed at the nuclear envelope.

Ultrastructural analysis of MO3.13 cells that overexpressed Lamin B1 showed well-preserved morphology, with regular shaped nuclei and nucleoli. The nuclear envelope was regularly composed of a double-layered membrane surrounding relaxed chromatin. Wild type and GFP-transduced MO3.13 cells showed similar morphology compared to Lamin B1 overexpressing cells, with polygonal shape morphology, well-preserved nucleus, nucleoli and the nuclear envelope regularly composed of two membrane bilayers.

#### Lamin B1 accumulation reduces both LIF and LIF-R expression

**Fig. 2:**
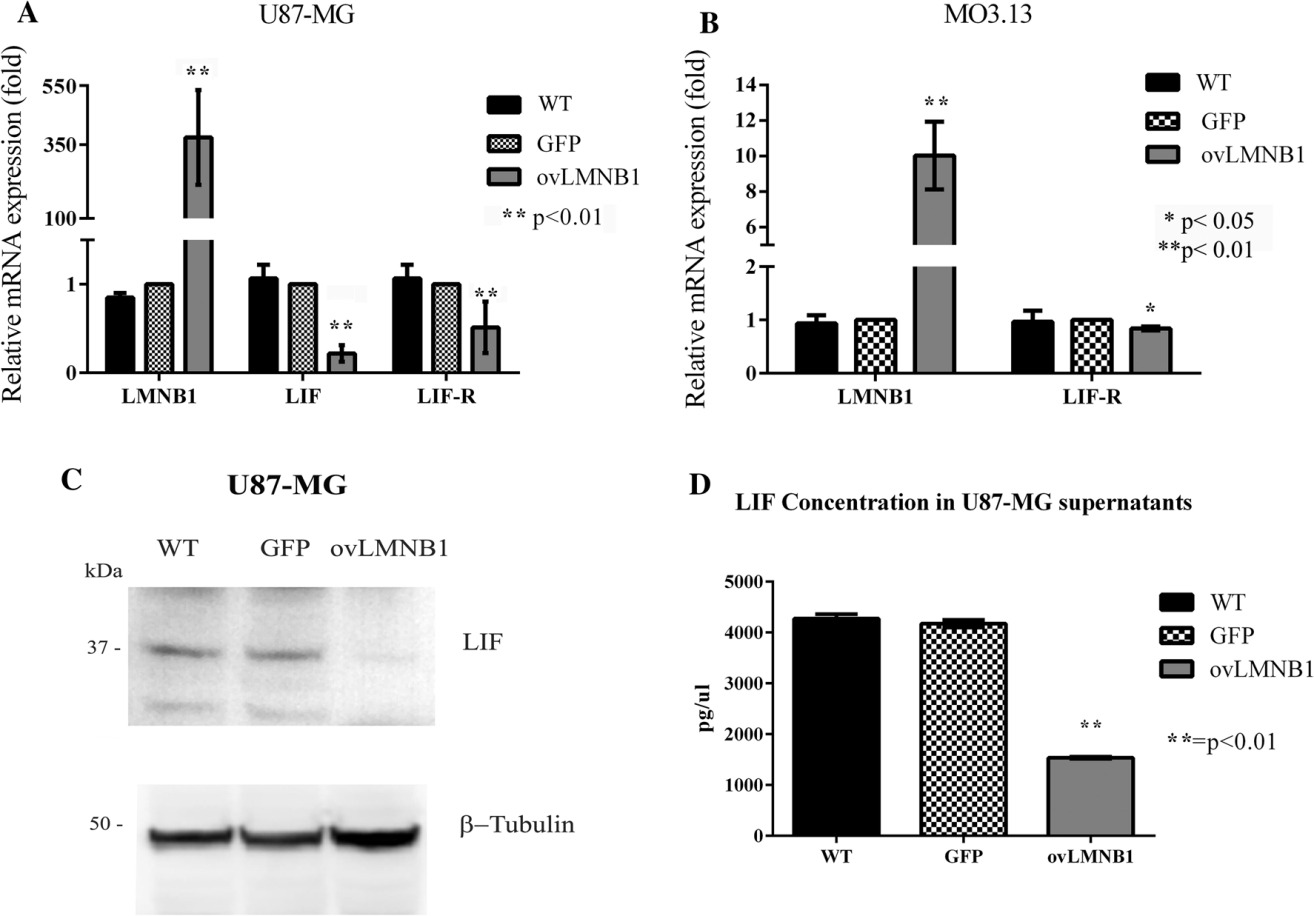
Lamin B1 accumulation reduces LIF and LIF-R expression. LIF and LIF-Receptor expression were tested in cells transduced to overexpress Lamin B1 (**ovLMNB1**) and compared to wild-type (**WT**) and mock-transduced (**GFP)** cells. Cells were tested after 48 h of puromycin selection, i.e. 96 h after transduction overall. U87-MG cells were tested for LIF and LIF-R mRNA levels by real-time PCR (**a**) and for LIF protein expression by western blot (**c**). GAPDH and β-tubulin were used as housekeeping gene and loading control, respectively. MO3.13 cells were tested for LIF-R mRNA levels by real-time PCR (**b**). Panel **d**: the supernatants of transduced U87-MG were tested for LIF concentration after puromycin selection. LIF concentration in Lamin B1 overexpressing sample (**ovLMNB1**) was compared to its concentration in wild type (**WT**) and mock-transduced (**GFP**) cells supernatants. All the analyses are from three independent experiments, with *p < 0.05 and ** *p* < 0.01 vs corresponding mock-transduced sample (GFP)

Given the role played by the leukemia inhibitory factor (LIF) in myelination and in the crosstalk between astrocytes and oligodendrocytes, we decided to evaluate the expression levels of both LIF and its receptor (LIF-R) in U87-MG cells, the astrocytes being the LIF-producing cells, and the expression of LIF-R in MO3.13 cells in response to Lamin B1 overexpression. In U87-MG cells, Lamin B1 accumulation resulted in a decrease of both LIF and LIF-R mRNA levels (Fig. 2a). In addition, in MO3.13 cells, we saw a significant reduction in LIF-R expression following Lamin B1 overexpression (Fig. 2b). We further evaluated LIF production in U87-MG cells by western blot and we confirmed the decrease in LIF expression following Lamin B1 overexpression (Fig. 2c). Next, we evaluated LIF concentration in the supernatants of wild type, mock-transduced and Lamin B1 overexpressing U87-MG cells. Figure 2d shows that LIF concentration was markedly reduced in the supernatants of Lamin B1 overexpressing cells; therefore, suggesting that the decrease seen at the mRNA and protein levels corresponded to an actual decrease in LIF release in the culture medium.

#### Lamin B1 build up affects the expression and phosphorylation of signaling molecules downstream LIF-R

Next, we investigated the signaling molecules involved in the pathways activated by LIF binding to LIF-R, *i.e.* PI3K/Akt, Jak/Stat3, and MEK/Erk pathways, to determine if the signaling cascades are active in Lamin B1 overexpressing cells.

**Fig. 3:**
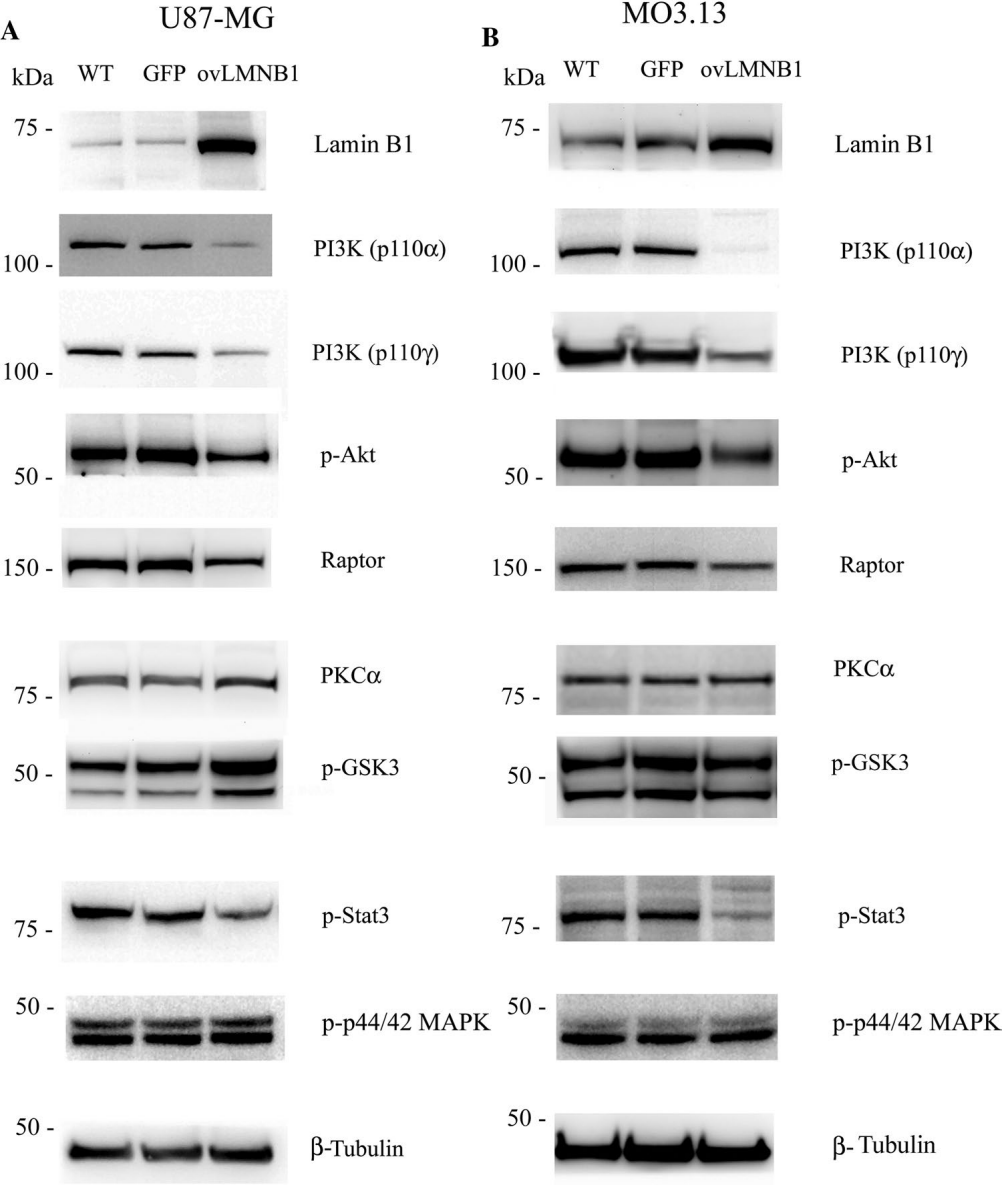
Lamin B1 build up affects the expression and phosphorylation of signaling molecules downstream LIF-R. The expression and the phosphorylation of molecules downstream LIF- signaling pathway was evaluated in U87-MG (**a**) and MO3.13 (**b**) cells 96 h after transduction. Lamin B1 overexpressing cells (**ovLMNB1**) were compared to wild type (**WT**) and mock-transduced (**GFP**) samples. The expression or phosphorylation of the denoted proteins was assayed. β-tubulin was used as loading control. Western blot results are representative of three independent experiments

Figure 3a shows that in U87-MG cells, Lamin B1 overexpression caused a down-regulation of the mediators belonging to the PI3K pathway. The amount of PI3K p110α and γ was both reduced as well as Akt phosphorylation and the expression of Raptor, a component of mTORC1 complex. However, the levels of phosphorylated GSK3 increased. This raise in GSK3 phosphorylation was paralleled by the increase in PKCα expression. On the other hand, Stat3 pathway was downregulated, confirmed by the reduction of Stat3 phosphorylation compared to wild type and mock-transduced cells (GFP). Nevertheless, the phosphorylation levels of p44/42 MAPK (Erk1/2) were not affected by Lamin B1 accumulation.

In MO3.13 cells, Lamin B1 overexpression also led to a reduction in PI3K activity as shown by the reduction in PI3K p110α and γ expression, Akt phosphorylation and the downstream target, Raptor expression. In contrast to the observation seen in U87-MG cells, GSK3 phosphorylation and PKCα expression did not change in MO3.13 cells overexpressing Lamin B1, but Stat3 signaling was downregulated just as observed in U87-MG cells. Lamin B1 accumulation did not alter Erk1/2 phosphorylation in MO3.13 cells either (Fig. 3b).

#### Evaluation of the effects of LIF administration on the downregulated signaling pathways

To investigate if LIF administration could revert the downregulation of the signaling pathways downstream LIF-R caused by Lamin B1 overexpression, 48 h after transduction cells were treated with 80 ng/ml LIF for 48 h. The activation of the downstream signaling pathways in Lamin B1 overexpressing cells was assayed by western blot analysis and compared to wild type and mock (GFP)-transduced cells.

**Fig. 4:**
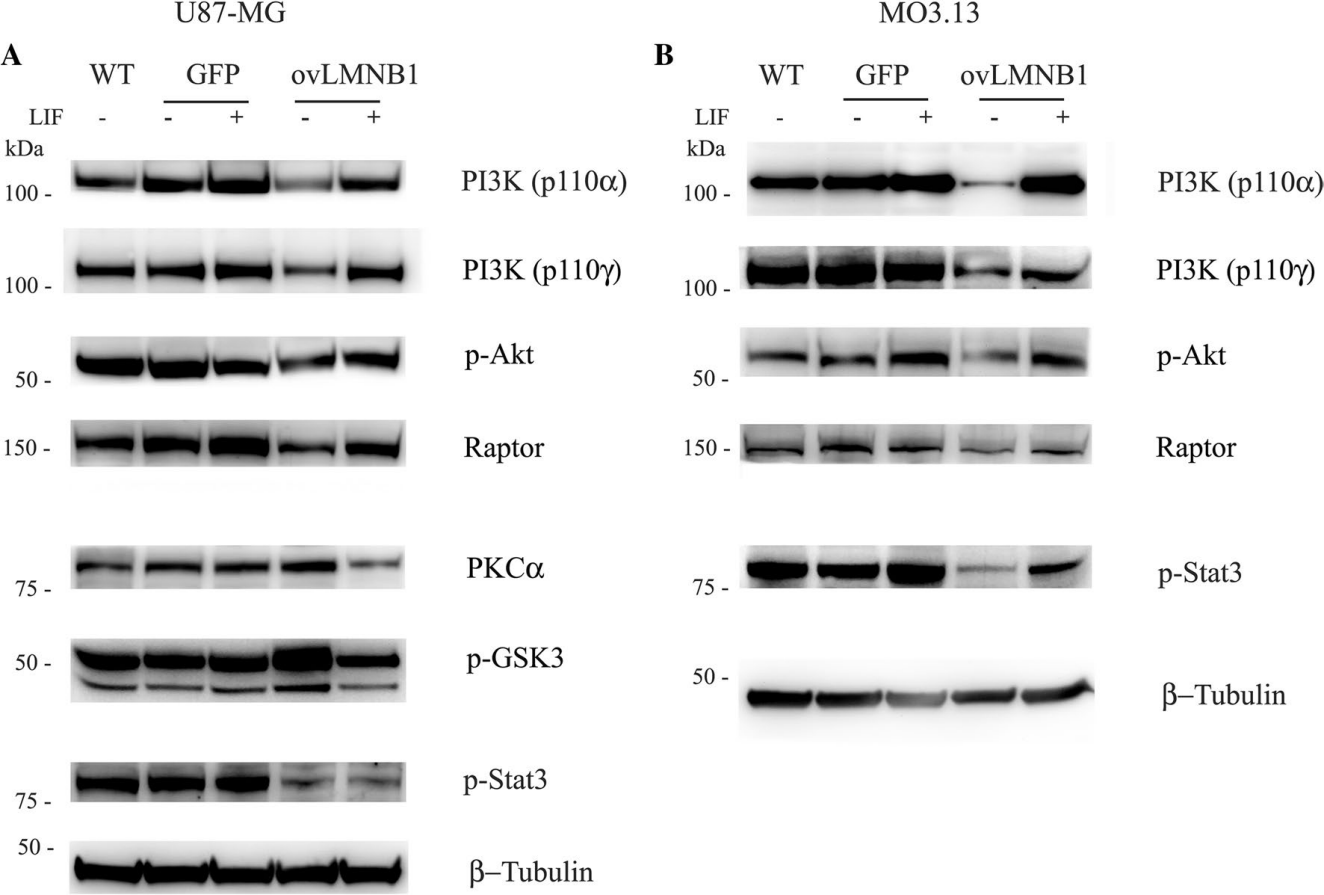
Effects of LIF treatment on downregulated signaling pathways. The effects of LIF administration were evaluated in U87-MG (**a**) and MO3.13 (**b**) cells following Lamin B1 overexpression (**ovLMNB1**) and compared to mock-transduced (**GFP**) and wild-type (**WT**) cells. Cells were transduced, puromycin-selected and either left in growth medium (−) or treated with LIF 80 ng/ml ( +) for 48 h. The expression or phosphorylation of the denoted proteins was assayed. β-tubulin was used as loading control. Western blot results are representative of three independent experiments

In U87-MG cells, the administration of LIF to Lamin B1 overexpressing cells resulted in the increase of PI3K p110α and γ, and Raptor expression, and Akt phosphorylation. Again, these proteins reached comparable levels to those seen in wild type and mock-transduced cells. In addition, in Lamin B1 overexpressing cells, PKCα expression and GSK3 phosphorylation were reduced in response to LIF treatment. Actually, PKCα expression was lower than in control cells. Nevertheless, no effect was detected on Stat3 phosphorylation that was lower in Lamin B1 overexpressing cells than in wild type and mock-transduced cells, even after LIF treatment (Fig. 4a). Therefore, LIF administration could partially revert the effect of Lamin B1 overexpression on mediators belonging to the PI3K pathway but it could not reactivate Stat3 signaling.

Since MO3.13 cells do not secrete LIF, we wanted to investigate if the stimulation with LIF could counteract the effects of Lamin B1 overexpression on the signaling pathways seen to be downregulated. LIF administration resulted in an increase of PI3K p110α and γ, and Raptor expression, and Akt phosphorylation in Lamin B1 overexpressing cells. Remarkably, in Lamin B1 overexpressing cells, Stat3 phosphorylation was re-activated in response to LIF administration even though it did not reach the levels of wild type and mock- transduced cells (Fig. 4b). Therefore, in MO3.13 cells, LIF administration could reverse almost completely the effects of Lamin B1 overexpression that were associated to a reduction in LIF-R expression.

### Ex vivo model: primary dermal fibroblasts from controls and ADLD patients

#### Lamin B1 accumulation affects the nuclear structure of dermal primary fibroblasts

**Fig. 5:**
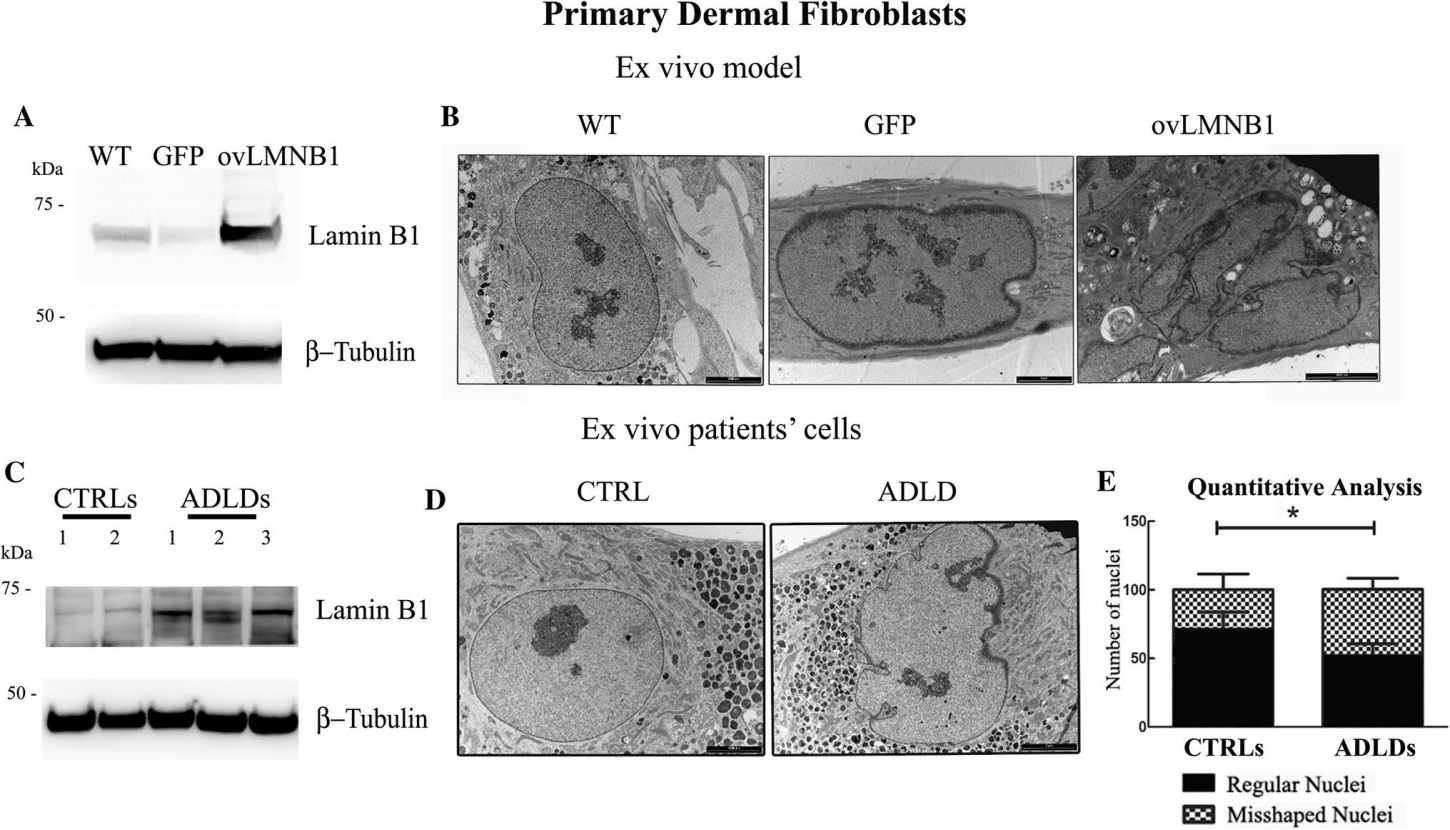
Ultrastructure of dermal primary fibroblasts isolated from healthy controls and ADLD patients. Primary dermal fibroblasts cultured from one healthy donor were transduced to overexpress Lamin B1. Lamin B1 expression was evaluated by western blot analysis, using β- tubulin as loading control. Western blot results are representative of three independent experiments (**a**). The ultrastructure of wild type, and GFP- and Lamin B1-overexpressing fibroblasts was evaluated by TEM (**b**). Control primary fibroblasts (**WT)** and of mock-transduced primary fibroblasts (**GFP**) have regular shaped nuclei and nuclear envelope (bar: 5000 nm and 1 μm, respectively). Dermal primary fibroblasts overexpressing Lamin B1 protein (**ovLMNB1**) have misshaped nuclei (bar: 5000 nm). The images are representative of three independent experiments. Lamin B1 expression was evaluated in primary dermal fibroblasts from healthy controls and ADLD patients by western blot analysis, using β-tubulin as loading control (**c**). Western blot results are representative of three independent experiments. The ultrastructure of fibroblasts from healthy controls and ADLD patients was evaluated by TEM (**d**). Dermal primary fibroblasts cultured from healthy patients (**CTRL**) show regular nuclear shape (bar: 5000 nm), while fibroblasts cultured from ADLD patients (**ADLD**) show misshaped nuclei (bar: 5 μm). The number of misshaped nuclei was compared to the number of regular nuclei observed in primary dermal fibroblasts cultured from controls (*n* = 6) and ADLD patients (*n* = 6) (**e**). Data are expressed in percentage, with *p < 0.05

Next, we evaluated the effects of Lamin B1 accumulation in fibroblasts by transducing dermal primary fibroblasts obtained from one healthy donor. This model was created to compare the effects of high levels of Lamin B1 expression in fibroblasts with the structural alterations seen in the engineered CNS cellular models. Therefore, dermal fibroblasts from one healthy donor were transduced with an empty vector (GFP) and with the vector coding for Lamin B1. Following the assessment of Lamin B1 overexpression by western blot (Fig. 5a), nuclear shape morphology was evaluated by TEM analysis (Fig. 5b). TEM analysis of transduced fibroblasts revealed that fibroblasts overexpressing Lamin B1 showed misshaped and irregular nuclei, while mock (GFP)-transduced and control dermal fibroblasts showed regular nuclear shape. The nuclear envelope of dermal fibroblasts that overexpressed Lamin B1 did not show any stacked lamellar membranes.

Additionally, Lamin B1 expression was assayed in fibroblasts from healthy donors and from ADLD patients by western blot (Fig. 5c). Generally, fibroblasts from ADLD patients showed higher Lamin B1 expression compared to healthy donors but its extent varied between samples from different patients. The ultrastructure of healthy and ADLD fibroblasts was also investigated by TEM (Fig. 5d). Dermal primary fibroblasts isolated from ADLD patients displayed more frequently irregular and misshaped nuclei compared to cells isolated from healthy donors. Quantitative analysis showed the presence of 48.03% of misshaped nuclei in ADLD patients compared to 28.6% in healthy donors (Fig. 5e). The percentage of misshaped nuclei in the healthy donors is related to the physiological transient and rapid nuclear shape changes due to the cell cycle activity [43]. These physiological alterations are also present in ADLD patients. Therefore, the significant difference of misshaped nuclei between healthy donors and ADLD patients is related to the pathology itself.

#### Lamin B1 accumulation induces the phosphorylation of inflammation mediators

Since fibroblasts are involved in chronic inflammation, we wanted to determine whether samples from healthy donors and from ADLD patients differed in the activation of inflammation mediators.

**Fig. 6:**
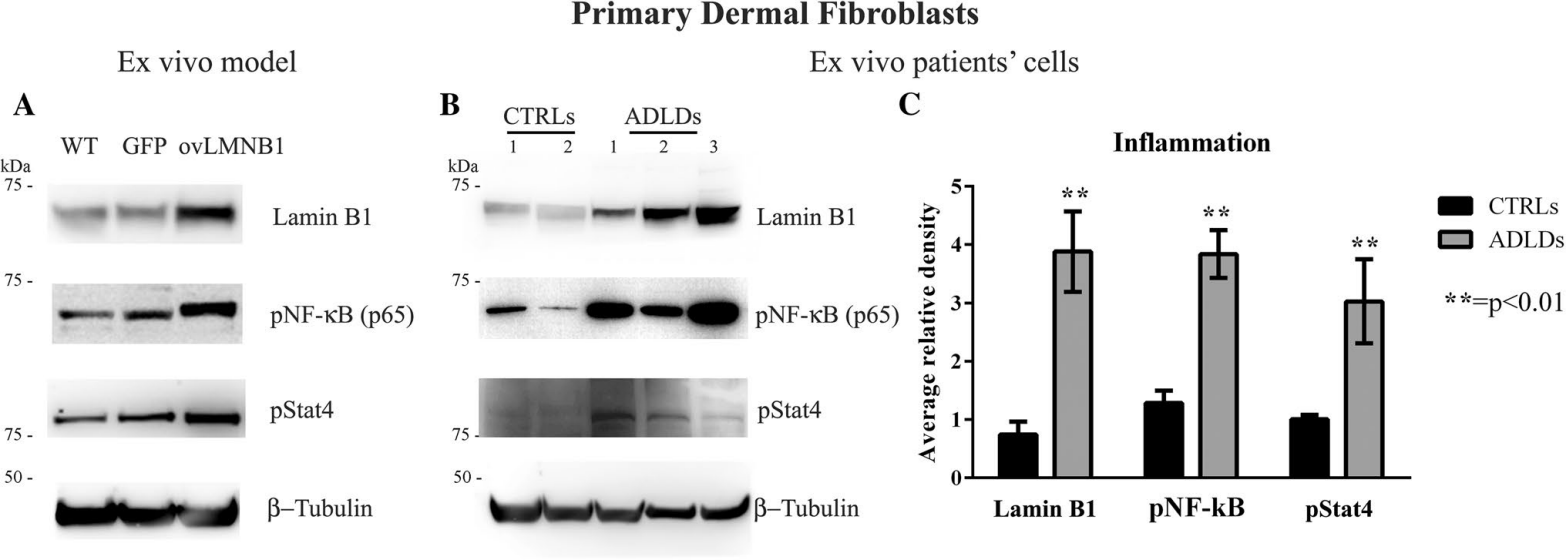
Lamin B1 overexpression induces the phosphorylation of inflammation mediators. Following transduction with mock (**GFP**) and Lamin B1 (**ovLMNB1**) coding vector, primary dermal fibroblasts from one healthy donor were evaluated for NF-kB and Stat4 phosphorylation and compared to wild type cells (**WT**) (**a**). **b** representative western blot showing NF-kB and Stat4 phosphorylation in fibroblasts from healthy donors (**CTRLs**) and ADLD patients (**ADLDs**). β-tubulin was used as loading control. **c** quantitative analysis of Lamin B1 expression, NF-kB and Stat4 phosphorylation in fibroblasts from healthy donors (**CTRLs**, *n* = 6) and ADLD patients (**ADLDs**, *n* = 6). Data are normalized to β-tubulin and are expressed as average ± SD. ** indicates *p* < 0.01 vs. control group

The phosphorylation levels of NF-kB and Stat4 were evaluated in fibroblasts from one healthy donor after transduction with an empty vector or with a vector coding for Lamin B1 and compared to wild type cells (Fig. 6a). Figure 6a shows that following Lamin B1 accumulation, transduced fibroblasts displayed higher levels of both NF-kB S536 and Stat4 Y693 phosphorylation. The association between Lamin B1 build up and NF-kB and Stat4 phosphorylation was evaluated also in primary dermal fibroblasts isolated from healthy donors and ADLD patients. Irrespective of the varied phosphorylation levels among different samples, as well as Lamin B1 expression levels, Fig. 6b shows that the amount of phosphorylated NF-kB and Stat4 was consistently higher in patients’ fibroblasts compared to controls. Statistical analysis of the densitometry values of the western blot bands showed a statistically significant difference in the phosphorylation levels between the two groups (Fig. 6c), suggesting that ADLD patients present higher activation of inflammation pathways compared to healthy donors.

#### ADLD patients produce more ROS in response to H_2_O_2_ treatment than healthy controls

**Fig. 7:**
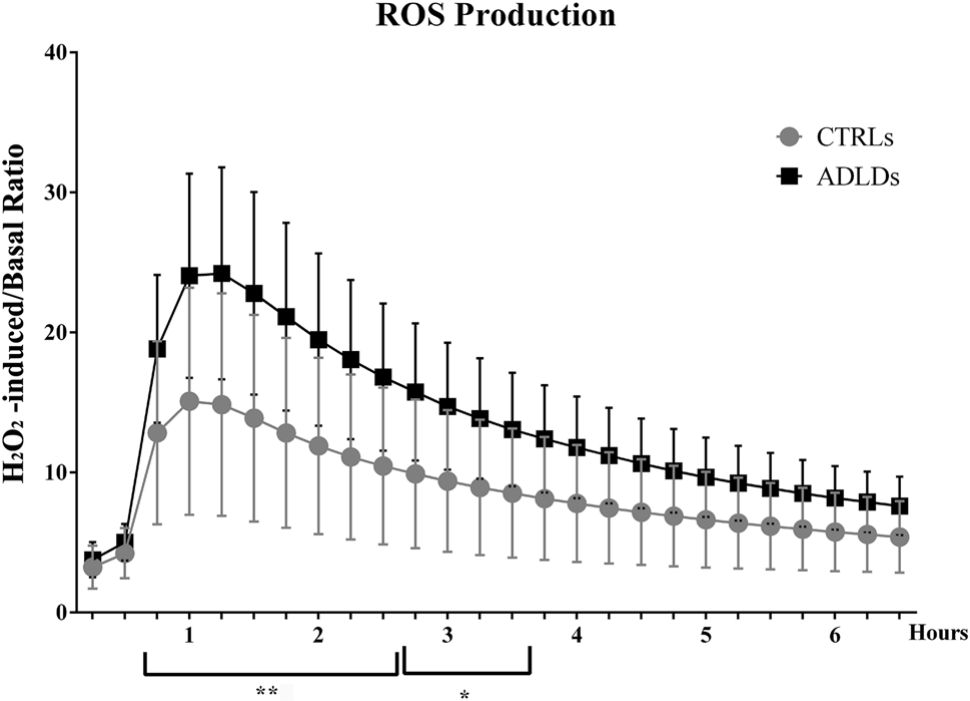
Fibroblasts’ ROS production in response to H_2_O_2_ treatment. The production of reactive oxygen species (ROS) in response to H_2_O_2_ treatment was evaluated in healthy donors (**CTRLs**, *n* = 6) and in ADLD patients (**ADLDs**, *n* = 6) every 15 mi for 7 h. Cells were loaded with 5 µM H2DCFDA probe, half of the sample was left untreated to measure basal ROS production, and the other half treated with 300 µM H_2_O_2_. Graph shows the ratio between H_2_O_2_- induced and basal ROS production during the time course. Analysis is from three independent experiments, with *p < 0.05 and ** *p* < 0.01 vs control group

To determine how fibroblasts from healthy donors and from ADLD patients respond to oxidative stress, we measured intracellular reactive oxygen species (ROS) production after H_2_O_2_ treatment at 15 min intervals for 7 h. Both groups showed an increase in ROS production after 45 min of stimulation, the production being higher in patients’ fibroblasts compared to controls (Fig. 7). The difference between the mean values detected in the two groups was statistically significant in the interval between 45 and 210 min after treatment, being highly significant (*p* < 0.01) between 60 and 135 min. Therefore, the assay shows that ADLD patients’ fibroblasts produce a higher amount of ROS in response to H_2_O_2_ stimulation.

## Discussion

ADLD is a rare adult-onset demyelinating neurological disease characterized by LMNB1 alterations and with no effective therapies. The patient phenotype described up to now mainly links the pathology to the overexpression of Lamin B1 protein due to LMNB1 duplication or to LMNB1 upstream deletion [44]. Nevertheless, recently it has been described that the mRNA level of LMNB1 from peripheral leukocytes of 2 Japanese patients of the same family with upstream deletion was not increased, maybe due to the differences in tissues used for mRNA examination [45]. Moreover, the clinical differences between ADLD patients with duplications and upstream deletions, such as the lack of autonomic dysfunction in the upstream deleted cases and the phenotypic differences between patients, underline that there are similar, but not identical pathological mechanisms underneath ADLD clinical, morphological, genetic and molecular phenotype. For this reason, the aim of this work was to highlight the significant morphological and cellular signaling alterations involved in ADLD, starting from patients with LMNB1 duplications and different engineered cellular models overexpressing Lamin B1 protein to define with more specificity the unknown mechanisms underneath the pathology.

**Fig. 8:**
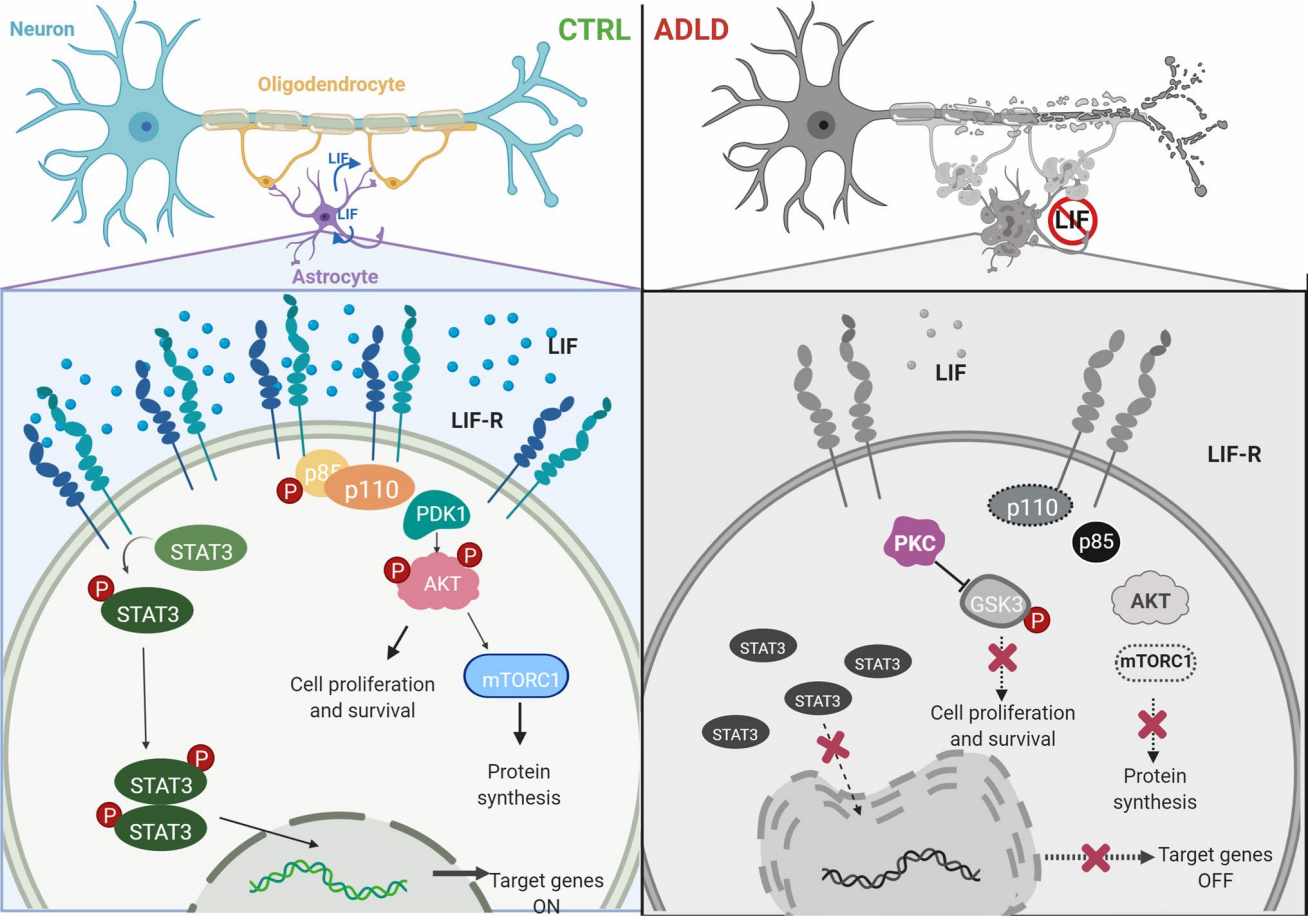
Astrocytes morpho-functional alterations in ADLD phenotype. In control astrocytes LIF pathway is active and involved in several physiological activities, in contrast with the morphological and signaling alterations highlighted in the ADLD model. In ADLD astrocytes, Lamin B1 accumulation causes alteration in the nuclear shape and impairment in Stat3 and PI3K/Akt axes associated to the activation of the PKC pathway: the structural suffering and the reduced support activity of astrocytes to oligodendrocytes determine alterations possibly involved in demyelination. Image created with Biorender.com

For the first time, the identification of new morphological and cellular signaling alterations has been linked to a specific CNS cellular type: the astrocyte. Indeed, our results related to cellular morphology show that severe ultrastructural nuclear alterations, such as misshaped and folded nuclei, are present in ADLD patients’ primary dermal fibroblasts, and both in control primary dermal fibroblasts and in the astrocyte cell line overexpressing Lamin B1, but not in the oligodendrocyte cell line overexpressing Lamin B1. Interestingly, the evidence of nuclear structure alterations, such as the increase of nuclear rigidity, has also been described in ADLD primary human skin fibroblasts and transient LMNB1 transfected HEK293 and neuronal N2a cells mimicking the mechanical phenotype of ADLD nuclei [46]. The correlation between these data and our new morphological evidence specifically on astrocytes, but not on oligodendrocytes points out a new relevant aspect: it is likely that different cellular types respond differently to the insult or toxicity related to the overexpression of Lamin B1. Considering the crucial role of oligodendrocytes in the myelination process and in ADLD pathogenesis [13], but also the controversial role that the oligodendrocyte specific Plp-FLAG-LMNB1 mouse model demonstrated in relation to the complexity of ADLD clinical phenotype and oligodendrocyte dysfunction [47], it is possible that the role of the astrocyte alteration might be pivotal in determining oligodendrocyte dysfunction, via cellular signaling mechanisms. The cellular suffering of Lamin B1 overexpressing astrocytes might, in fact, find more relevant and functional explanations if we consider the molecular mechanisms altered in this engineered cell line. In these cells, Lamin B1 severe accumulation induces a reduction in LIF and in LIF-R levels with a consequential decrease in LIF production and its extracellular release. Moreover, oligodendrocytes overexpressing Lamin B1, that cannot physiologically produce LIF, show a reduction in LIF-R expression. Therefore, the communication between astrocytes and oligodendrocytes might be altered by the reduction of the “active” role of the astrocytes and the reduction of the “passive” role of the oligodendrocytes regarding LIF signaling, crucial in the myelination processes [25]. In both our engineered cellular models overexpressing Lamin B1, Jak/Stat3 and PI3K/Akt axes, downstream of LIF/LIF-R, are downregulated, instead the third pathway associated to LIF/LIF-R, MEK/Erk axis, is not modulated. The reason of these results might be found in the fact that Jak/Stat3 pathway has a more direct connection to LIF axis, whereas several signaling pathways converge and interact with both PI3K/Akt and MEK/Erk axes. Very interestingly, in Lamin B1 overexpressing U87-MG cells, GSK3 phosphorylation is paralleled by the increase in PKCα expression, suggesting that this kinase might be responsible for GSK3 phosphorylation, whereas in Lamin B1 overexpressing MO3.13 cells no variation in its phosphorylation can be observed. This specificity might be involved in several cell proliferation and survival mechanisms that are more impaired in ADLD-like astrocytes. The addition of nuclear membrane alterations and cellular signaling deficiencies might explain some of the pathological aspects correlated to ADLD [44, 48] (Fig. 8). Significantly, the phenotype rescue experiments show that the administration of exogenous LIF can partially reverse the toxic effects induced by Lamin B1 overexpression correlated to the downregulation of LIF axis with differences between astrocytes and oligodendrocytes. Specifically, the administration of LIF determines a rescue in the protein expression of PI3K p110α and γ, Raptor and Akt phosphorylation in both Lamin B1-overexpressing U87-MG and MO3.13 cells. Moreover, PKCα expression and GSK3 phosphorylation are reduced in response to LIF treatment in Lamin B1 overexpressing U87-MG cells. It is possible that the treatment-induced reduction of PKCα is indeed involved in the reduced phosphorylation of GSK3; therefore, its re-activation that may be involved in promoting cell survival. PKCα appears to be reduced in LIF treated Lamin B1 overexpressing U87-MG also in comparison with wild type cells probably due to a physiological compensation role in response to the great activation following Lamin B1 accumulation. Finally, LIF administration upregulates Stat3 phosphorylation, but only in Lamin B1 overexpressing MO3.13 cells, underlying that it is possible to restore normal cellular pathways in oligodendrocytes, probably less affected by Lamin B1 toxicity, bypassing LIF-R downregulation with exogenous administration. On the other hand, astrocyte signaling can only be restored partially by LIF, as Stat3 phosphorylation cannot be reestablished; therefore, highlighting that Lamin B1 overexpression drastically affects astrocytic function by reducing the pivotal support to the oligodendrocytes in the myelination process. It is possible that the reduction of both LIF and LIF-R expression in astrocytes, induced by Lamin B1 accumulation, creates a negative autocrine loop in this cell type that impairs its functional characteristics. Instead, oligodendrocytes are not affected morphologically by Lamin B1 accumulation and can benefit almost completely by LIF administration, as if their alterations in ADLD might be consequential and secondary to the alterations induced to astrocytes. Indeed, LIF can regulate astrocytes maturation, activation, and oligodendrocytes differentiation from progenitor cells. LIF production can be regulated by many different factors such as interleukins under different conditions in different tissue/cell types [49] and it has been investigated also in relation to Multiple sclerosis (MS) as a potential therapeutic factor [50] and to neuroinflammatory lesions [51]. Given that astrocytes can produce LIF in vitro [52], LIF receptor signaling can limit immune-mediated demyelination by enhancing oligodendrocyte survival [53, 54]. For this reason, and considering the involvement of fibroblasts in chronic inflammation, we also investigated the role of inflammation in ADLD primary dermal fibroblasts, to add another piece of information to the ADLD intriguing puzzle. Interestingly, ADLD primary dermal fibroblasts show an increase in the phosphorylation of important inflammation mediators, such as NF-kB and Stat4, and an increase in ROS production in response to H_2_O_2_, compared to the healthy donors. Indeed, oxidative stress has been investigated in relation to different cell proliferation and age dependent alterations linked to Lamin B1 accumulation [55]. It is possible that the supposed correlation between metabolic alterations, inflammation and stress age-related processes [44] might reinforce the pathological mechanisms underneath the progression of ADLD phenotype, inducing a negative loop mechanism. Concluding, given that the aberrant cellular mechanisms are evident in various neurological disorders [56] and that Lamin B1 has a central and complicated role in cell cycle regulation [57, 58], the alteration of signaling pathways might represent an important event underlying the disease phenotype. Considering the recent efforts in finding possible therapeutic strategies for ADLD [59], the comprehension, for the first time, of specific astrocyte cellular mechanisms linked to morphological alteration underneath this pathology might pave the way to new relevant ADLD investigation perspectives.
